# Intermedin Reduces Oxidative Stress and Apoptosis in Ventilator-Induced Lung Injury via JAK2/STAT3

**DOI:** 10.3389/fphar.2021.817874

**Published:** 2022-01-24

**Authors:** Shulei Fan, Jing He, Yanli Yang, Daoxin Wang

**Affiliations:** Department of Respiratory Medicine, Second Affiliated Hospital of Chongqing Medical University, Chongqing, China

**Keywords:** intermedin (IMD), ventilator-induced lung injury, oxidative stress, apoptosis, JAK-STAT signaling pathway

## Abstract

Mechanical ventilation is an effective treatment for acute respiratory distress syndrome (ARDS), which can improve the prognosis of ARDS to a certain extent. However, it may further aggravate lung tissue injury, which is defined as ventilator-induced lung injury (VILI). Intermedin (IMD) belongs to the calcitonin gene-related peptide (CPRP) superfamily. Our previous studies have found that IMD reduces the expression proinflammatory cytokines, down-regulates nuclear translocation and improves the integrity of endothelial barrier in ARDS. However, the effect of IMD on VILI has not been clarified. Oxidative stress imbalance and apoptosis are the main pathophysiological characteristics of VILI. In the current study, we used C57B6/J mice and human pulmonary microvascular endothelial cells (HPMECs) to establish a VILI model to analyze the effects of IMD on VILI and explore its potential mechanism. We found that IMD alleviated lung injury and inflammatory response in VILI, mainly in reducing ROS levels, upregulating SOD content, downregulating MDA content, reducing the expression of Bax and caspase-3, and increasing the expression of Bcl-2. In addition, we also found that IMD played its anti-oxidative stress and anti-apoptotic effects via JAK2/STAT3 signaling. Our study may provide some help for the prevention and treatment of VILI.

## Introduction

Acute respiratory distress syndrome (ARDS) is one of the most common critical diseases of the respiratory system, with high mortality and a heavy social burden (Matthay et al., 2019). Mechanical ventilation (MV) restores respiratory muscles and provides sufficient gas exchange, which improves the prognosis of ARDS to a certain extent (Slutsky and Ranieri, 2013; Gaver et al., 2020). However, mechanical ventilation may aggravate lung tissue injury, mainly manifested in infiltration of neutrophils, formation of hyaline membrane, increase of apoptosis, up-regulation of vascular endothelial permeability and formation of pulmonary edema, which is called ventilator-related lung injury (Curley et al., 2016). Endothelial cells (ECs) are the earliest effector cells in lung injury (Matthay et al., 2019). Therefore, reducing endothelial cell injury and maintaining endothelial cell function is the key to alleviating endothelial permeability, reducing pulmonary edema, and reducing mortality (Qi et al., 2016; Huang et al., 2017).

Intermedin (IMD) is a novel polypeptide belonging to the calcitonin superfamily. IMD is widely expressed throughout the body, and its sequence is highly conserved among different species (Hong et al., 2012; Holmes et al., 2020; Sohn et al., 2020). IMD is a homeostasis regulating peptide involved in many life activities, such as glucose and lipid metabolism, inhibition of inflammation, maintaining cardiovascular system stability, tissue repair, and other aspects (Wang et al., 2020; Ren et al., 2021). Our previous study found that IMD reduced inflammation, improved pulmonary microvascular endothelial cell barrier integrity and function, eased pulmonary edema, and alleviated vascular leakage in LPS-induced ARDS (Fan et al., 2020). However, the effects of IMD on VILI is unclear.

In the present study, we used 18% cyclic stretching (CS)-induced human pulmonary microvascular endothelial cells (HPMECs) and mechanical ventilation (MV)-induced C57BL/6J mice as VILI models to clarify the anti-apoptotic and anti-oxidative stress effects of IMD. Our study may provide some help for the prevention and treatment of VILI.

## Materials and Methods

1. VILI animal model

Healthy C57BL/6J mice (8–10-week-old, male) were purchased from the Experimental Animal Center of Chongqing Medical University (Chongqing, China). All mice were housed at a 12/12 h dark/light cycles and were allowed access to food and water. Establishment of the VILI mouse model (Yu et al., 2020): After endotracheal intubation, an animal ventilator (Alcott Biotechnology, Shanghai, China) for MV was calibrated as follows: the tidal volume was 30 ml/kg, the respiratory rate was 75 times/min, the positive end expiratory pressure (PEEP) was 0 cmH_2_O, and the duration of MV was 4 h. IMD1-53 (the active fragment of IMD peptide, containing 53 amino acids) (50 ng/kg) or an IMD inhibitor [IMD17-47, a truncated fragment of IMD peptide with 31 amino acid residues, that was used as the competitive inhibitor of IMD (Xiao et al., 2018; Zhang and Xu, 2018)] (100 ng/kg) was subcutaneously injected 1 h before the MV. IMD1-53 and IMD inhibitor (IMDinh) were purchased from Phoenix Pharmaceuticals (CA, United States). In some experiments, the JAK2/STAT3 agonist Colivelin TFA (MedChemExpress, Shanghai, China, 1 mg/kg) was injected intraperitoneally into the mice 30 min before the IMD1-53 injection. All *in vivo* experimental procedures were performed in accordance with the guidelines for the Care and Use of Laboratory Animals by the National Institutes of Health, and were approved by the Ethics Committee of the Second Affiliated Hospital of Chongqing Medical University (Chongqing, China).

2. Cell culture

HPMECs (from ScienCell) were cultured in EC medium containing 1% EC growth supplement, 10% fetal bovine serum and 1% penicillin–streptomycin in an incubator. Cyclic stretching (CS) was performed for 4 h to establish EC models (18% CS for VILI model and 5% CS for the spontaneous breathing model) using a Flexcell FX-4000 Tension System (Flexcell, Burlington, United States) (Yu et al., 2020). In addition, HPMECs were cultured in IMD1-53 (10^−7^ mol/L) or IMDinh (10^−6^ mol/L) 1 h before CS. In some experiments, which detected the potential effects of the JAK2/STAT3 pathway in IMD-mediated protection, Colivelin TFA (MedChemExpress, Shanghai, China, 1 μM) was administrated half an hour before IMD1-53 pretreatment.

3. Hematoxylin and eosin (H&E) staining and lung histology injury evaluation

The mice lung tissues were soaked in 4% paraformaldehyde. Next, the specimens were embedded in paraffin, cut into sections and stained with hematoxylin and eosin (H&E). The stained slides were analyzed using a microscope (Olympus, Tokyo, Japan). The lung injury score was measured as previously described (Wang et al., 2014).

4. Measurement of SOD and MDA

The levels of SOD and MDA were measured using SOD or MDA assay kit (Solarbio, Beijing, China) followed by the manufacturer’s instructions.

5. Immunohistochemistry

Lung tissue sections from the left mice were deparaffinized with xylene, rehydrated in ethanol, and incubated in 3% H_2_O_2_ for 15 min. Then sections were washed and then blocked with goat serum for 1 h and incubated overnight at 4°C with the anti-primary antibody (Bax, caspase-3 and Bcl-2, all from servicebio, Hubei, China). Then, the sections were washed and incubated with secondary antibody (servicebio, Hubei, China) for 30 min, and then stained with DAB solution. Sections were counterstained with hematoxylin, dehydrated, vitrified and sealed.

6. Flow cytometry for reactive oxygen species (ROS)

An ROS detection kit (Beyotime, Shanghai, China) was used to detect ROS levels. The ECs were cultured in DCFH-DA solution at 37°C for 20 min, washed with serum-free cell culture medium. Then, the cell suspensions were collected. Finally, ROS levels were analyzed using a flow cytometer (Beckman, Georgia, United States).

7. Quantitative real-time PCR (qRT-PCR)

Total RNA was isolated from the cells and mice lung tissues using RNAiso Plus (Takara, Beijing, China). Then, 1 μg of total RNA was used as a template for amplifying the cDNA using the PrimeScript™ RT reagent kit (Takara, Beijing, China). cDNA was used for qPCR amplification using TB Green™ Premix Ex Taq™ II (Takara, Beijing, China). All data were calculated using the 2-ΔΔCt method and normalized to β-actin. Primer sequences for the mice were as follows: Bax (sense:5′- CCTCCTCTCCTACTTTGGGAC-3′, antisense:5′-GAAAAACACAGTCCAAGGCAG-3′); caspase-3 (sense:5′- GGACTCTGGAATATCCCTGGAC-3′, antisense:5′- TTTGCTGCATCGACATCTGTAC-3′); Bcl-2 (sense:5′- GTGGCCTTCTTTGAGTTCGG-3′, antisense:5′- GGTGCCGGTTCAGGTACTCA-3′); IL-6 (sense 5′- ATGAGGAGACTTGCCTGGTGAA -3′, antisense 5′- GTTGGGTCAGGGGTGGTTATT-3′); TNF-α (sense 5′- TCATCTACTCCCAGGTCCTCTTCA-3′, antisense 5′- TCTGGCAGGGGCTCTTGATG-3′); β-Catenin (sense: 298 5′-GTTCTACGCCATCACGACACTG-3′, antisense: 5′-TTGCTCTCTTGATTGCCATAAGC-3′).

8. Western blotting

Total proteins were extracted from tissues and ECs using RIPA, and the concentration was detected by BCA kit (Solarbio, Beijing, China). Equal amounts of protein were loaded into each well for separation via SDS-PAGE and electrophoresed onto PVDF membranes. The membranes were blocked with 5% skim milk or 5% BSA for 1 h, incubated with primary antibodies (Bax, caspase-3, Bcl-2, JAK2, p-JAK2, STAT3, p-STAT3 and β-actin) (β-actin purchases from Bioworld Technology, Nanjing, China, and others from Abcam, Cambridge, United Kingdom) overnight at 4°C, and then incubated with secondary antibodies (Bioworld, Nanjing, China). Finally, the protein bands were measured using the ECL Substrate kit (BioGround Biotechnology, China) and images were captured using Imaging System (Bio-Rad, CA, United States).

9. ROS staining

Fresh lung tissues were embedded in the OTC and sliced. The sections were rewarmed to room temperature while controlling the moisture content, after which an autofluorescence quenching agent was added and cleaned. Thereafter, the sections were stained with ROS dye and kept away from light for 30 min. Finally, they were counterstained with DAPI, sealed, and observed under a fluorescence microscope.

10. Flow cytometry

An apoptosis detection kit was used to access cell apoptosis. All processes were in accordance with the manufacturer’s requirements. A flow cytometer (Beckman, Georgia, United States) was used to analyzed the data.

11. Statistical analysis

Continuous data are displayed as the mean ± standard deviation (SD). T-test or ANOVA is performed to detected significance between groups if the data are normally distributed, while the Mann-Whitney U test or Kruskal-Wallis test is used if the data are non-normally distributed. To assess subgroup analysis, Tukey test or Dunn’s multiple comparisons test is measured. The chi-squared test is used to compare categorical variables between groups. Statistical significance is set at *p* < 0.05.

## Results

1. IMD attenuated lung injury and inflammation in VILI

**FIGURE 1:**
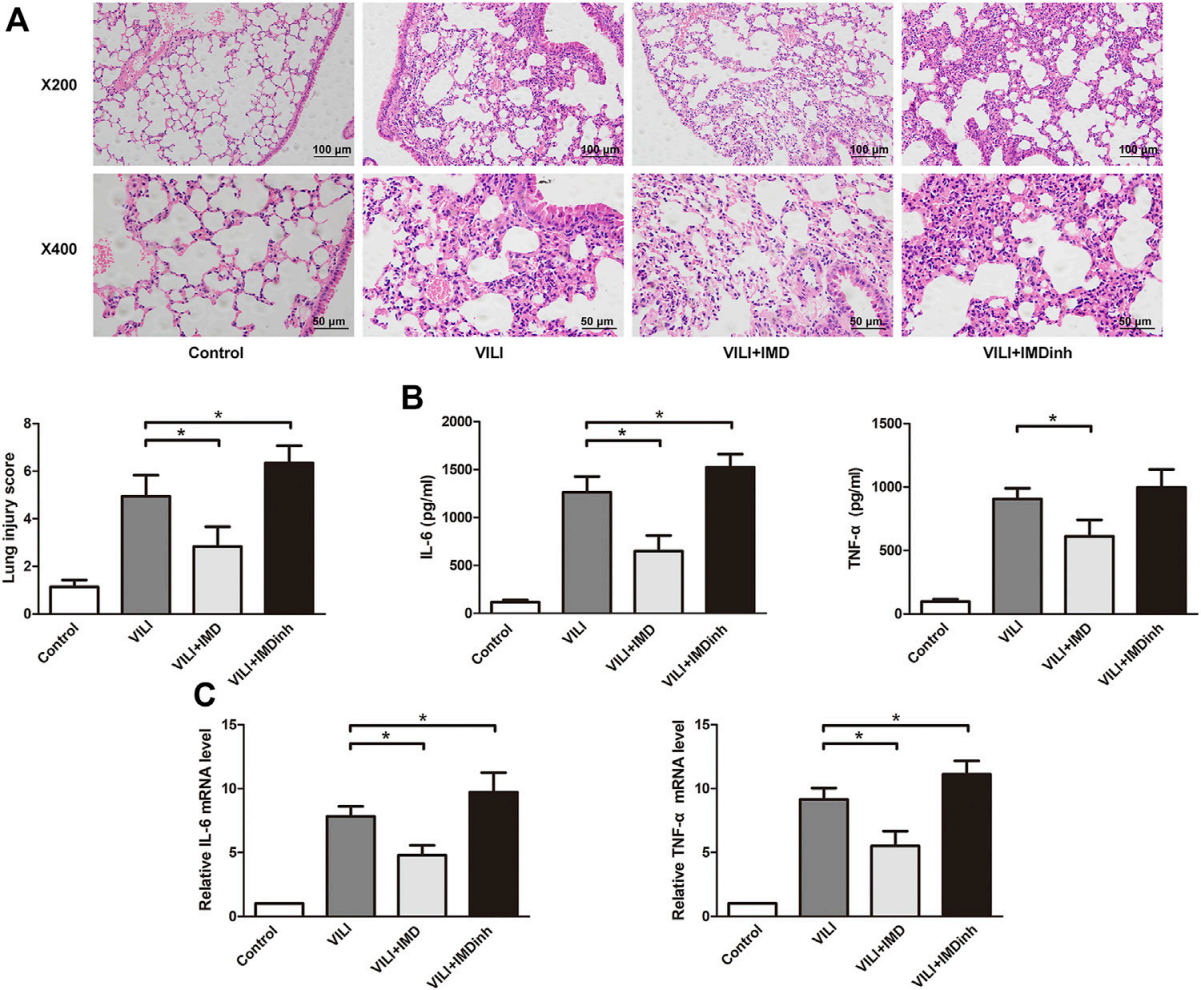
IMD alleviated lung injury inflammation *in vivo* and *in vitro*. **(A)** H&E staining (×200 and ×400), lung injury score was used to evaluate lung histopathological injury. **(B)** The concentration of IL-6 and TNF-α in mice lung tissues. **(C)** The expression of IL-6 mRNA and TNF-α in HPMECs. *p < 0.05.

*In vivo*, IMD or IMDinh (inhibitor of IMD) was administered 1 h before MV and then MV was administered for 4 h. H&E and lung injury score results showed that IMD pretreatment significantly attenuated lung tissue injury caused by MV, mainly manifesting as narrowing of the alveolar septum, reduction of neutrophil infiltration, perivascular edema, protein edema fluid exudation, and patchy bleeding. IMDinh was truncated structure of IMD, which was worked as a competitive inhibitor of IMD. It inhibited the biological functions of endogenous IMD and aggravated lung tissue injury caused by MV (Figure 1A). ELISA for IL-6 and TNF-α of lung homogenates showed that IMD pretreatment significantly reduced the increase in IL-6 and TNF-α levels induced by MV. However, IMD inhibitor played the opposite role (Figures 1A–C). *In vitro*, HPMECs were subjected to 18% CS (VILI model) or 5% CS (spontaneous breathing, control model) for 4 h. IMD or IMDinh was given 1 h before 18% CS. PCR results showed that compared with the VILI group, the mRNA levels of IL-6 and TNF-α in the VILI + IMD group were significantly alleviated. However, IMDinh further exacerbated the mRNA levels of proinflammatory cytokines (IL-6 and TNF-α) (Figures 1D,E). The above data demonstrated that IMD attenuated lung injury and inflammation both *in vivo* and *in vitro*.

2. IMD alleviated apoptosis in mice and in ECs

**FIGURE 2:**
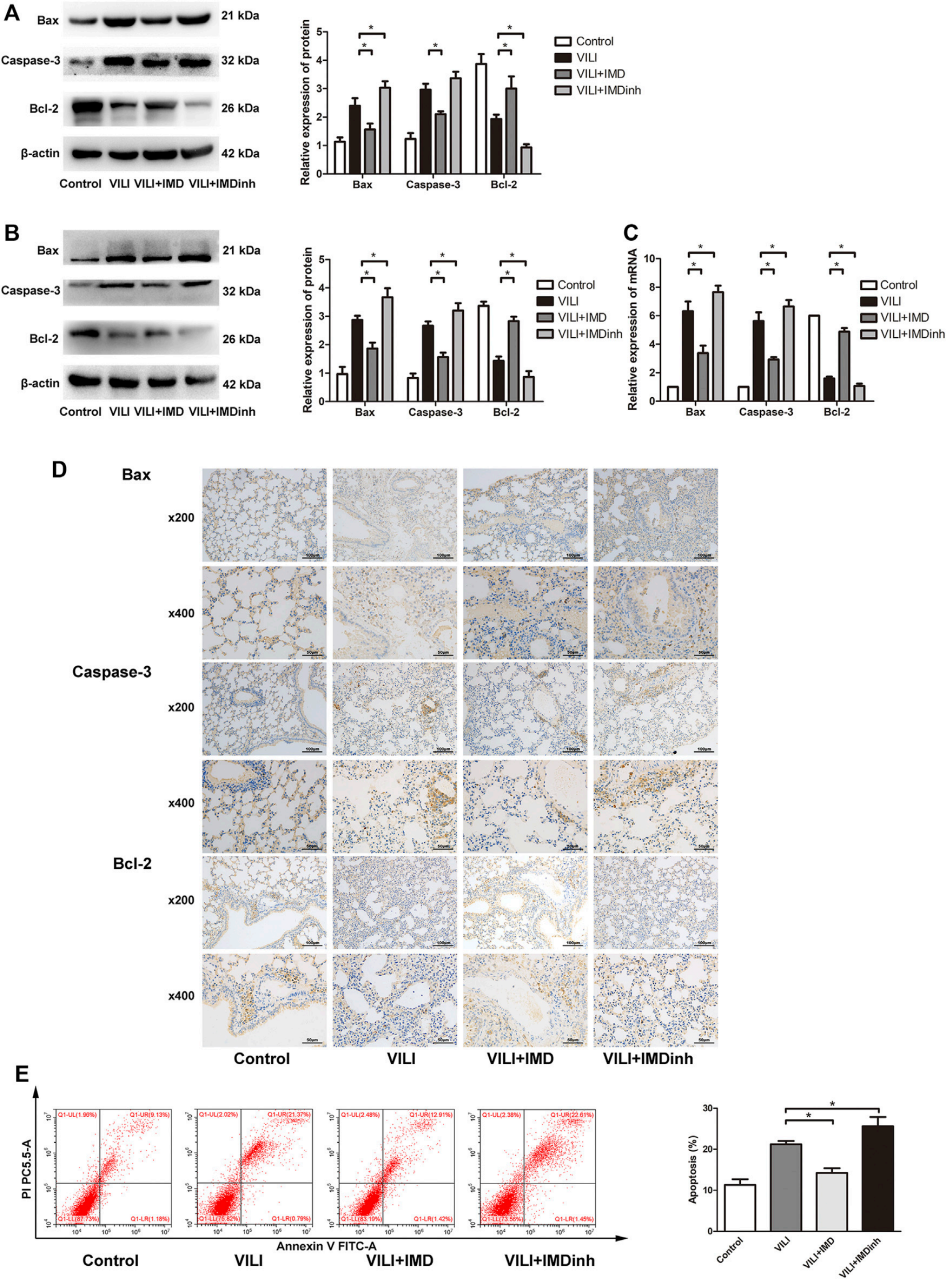
IMD alleviated apoptosis in mice and in ECs. Western blot analysis of Bax, caspase-3, Bcl-2 and β-actin in lung tissues **(A)** and ECs **(B)**. **(C)** Immunohistochemistry of Bax, caspase-3, Bcl-2 and β-actin in lung tissues. **(D)** Cell apoptosis rate was detected using flow cytometry. *p < 0.05.

The expression of apoptosis related protein (Bax, caspase-3, and Bcl-2) was detected by western blotting, PCR, and immunohistochemistry to evaluate the anti-apoptotic role of IMD in VILI. Western blotting and qPCR showed that in the VILI model group, the expression of Bax and caspase-3 increased significantly while the expression of Bcl-2 decreased significantly (Figures 2A–C). However, compared with the VILI group, IMD pretreatment significantly reduced the expression of Bax and caspase-3 and increased the expression of Bcl-2 (Figures 2A–C). Immunohistochemistry showed a significant alleviation of histopathologic damage in the lungs after IMD treatment (Figure 2D). We also found that compared with the VILI group, if IMD pretreatment was given in advance, it could significantly reduce the expression of Bax and caspase-3 and increase the expression of Bcl-2. In addition, flow cytometry analyses demonstrated that the administration of IMD voided ECs from apoptosis (Figure 2E). However, IMDinh displayed the opposite effect (Figures 2A–E). These findings indicated that IMD reduced ventilator-induced apoptosis *in vivo* and *in vitro*.

3. IMD alleviated oxidative stress

**FIGURE 3:**
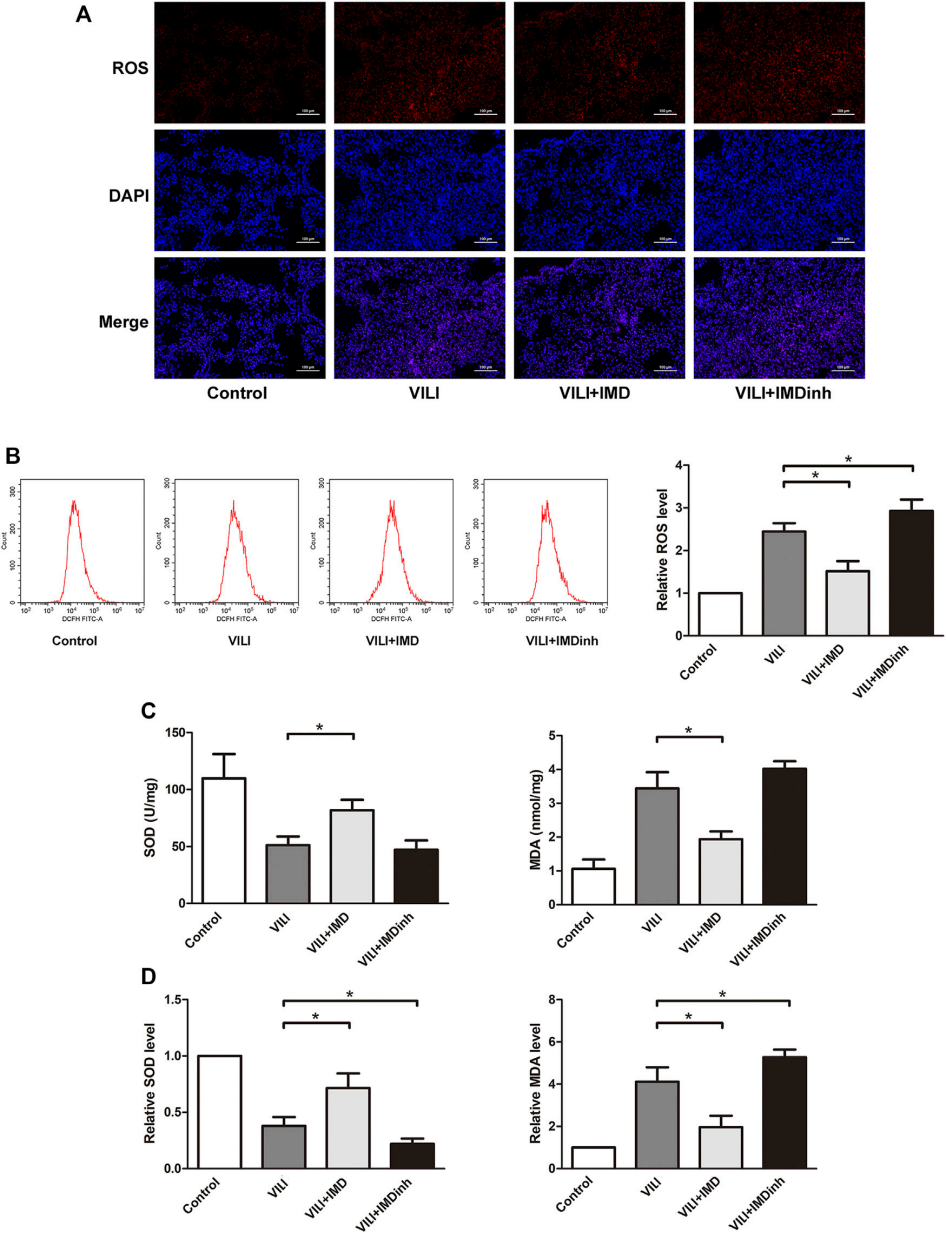
IMD alleviated oxidative stress in mice and in ECs. **(A)** ROS staining of mice lung tissues. **(B)** Cell ROS was detected using flow cytometry. **(C)** The expression of SOD and MDA in mice lung tissues was measured by ELISA. **(D)** The expression of SOD and MDA in ECs was measured by ELISA. *p < 0.05.

Excessive oxidative stress generated by injured ECs is widely involved in the progression of lung damage during VILI (Fodor et al., 2021). The imbalance of ROS is the main driving factor for oxidative stress (Piera-Velazquez and Jimenez, 2021). In mice, frozen sections of the lungs were prepared, and ROS were measured by detecting the fluorescence intensity of O_13_. We found that IMD significantly reduced the generation of ROS in the VILI group (Figure 3A). In HPMECs, ROS was measured by detecting the fluorescence intensity of DCF, and we found that IMD significantly alleviated the generation of ROS induced by VILI (Figure 3B). SOD and MDA are important indices for evaluating the antioxidant and oxidative capacities of oxidative stress (Fodor et al., 2021). *In vivo* and *in vitro*, compared with the VILI group, IMD pretreatment significantly increased SOD levels and decreased MDA levels (Figures 3C,D). However, IMDinh had the opposite effect and aggravated oxidative stress. These data demonstrated that IMD alleviated oxidative stress *in vivo* and *in vitro*.

4. JAK2/STAT3 pathway was involved in IMD-mediated protection

**FIGURE 4:**
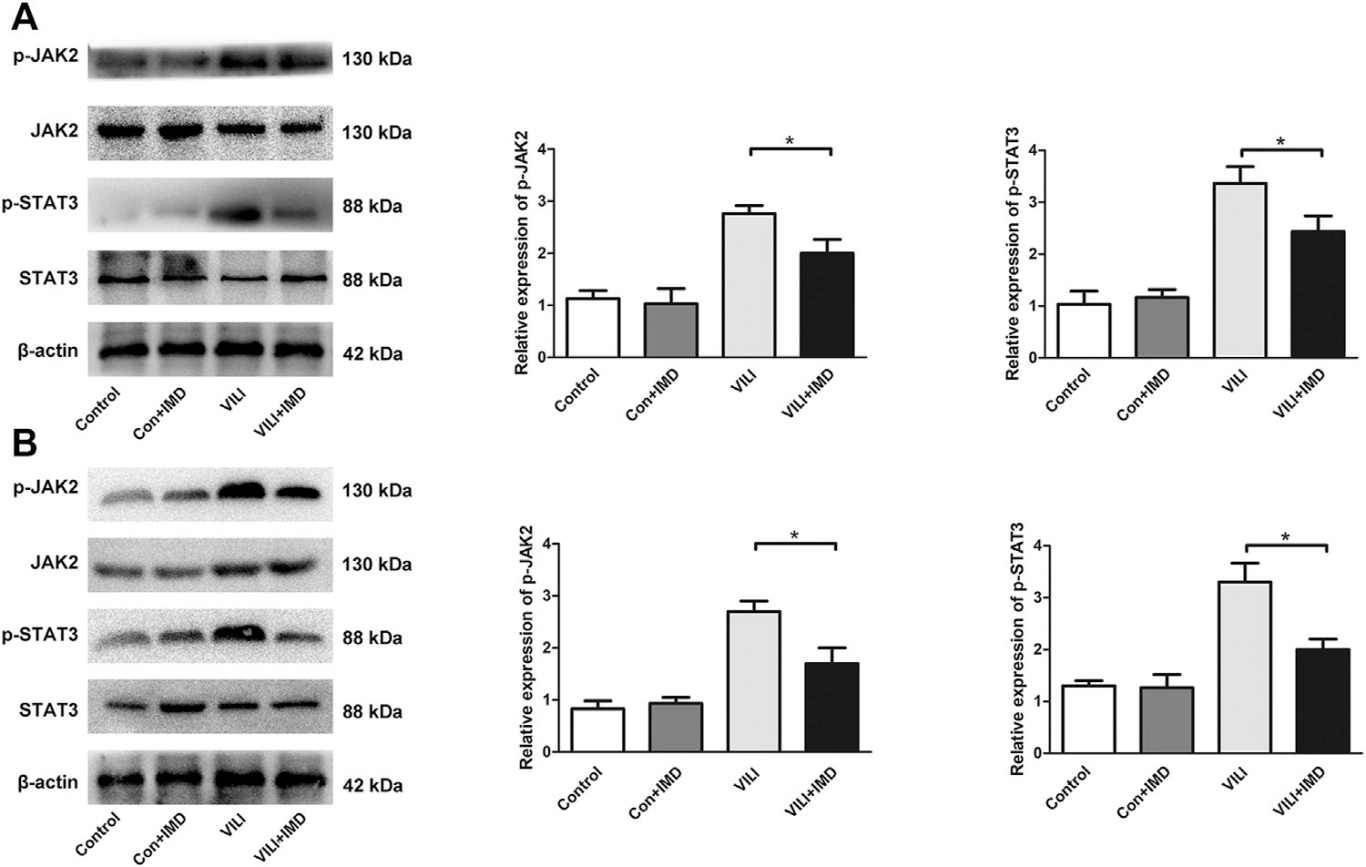
IMD inhibited the JAK2/STAT3 pathway in VILI. Western blot analysis of p-JAK2, JAK2, p-STAT3, STAT3 and β-actin in lung tissues **(A)** and ECs **(B)**. *p < 0.05.

**FIGURE 5:**
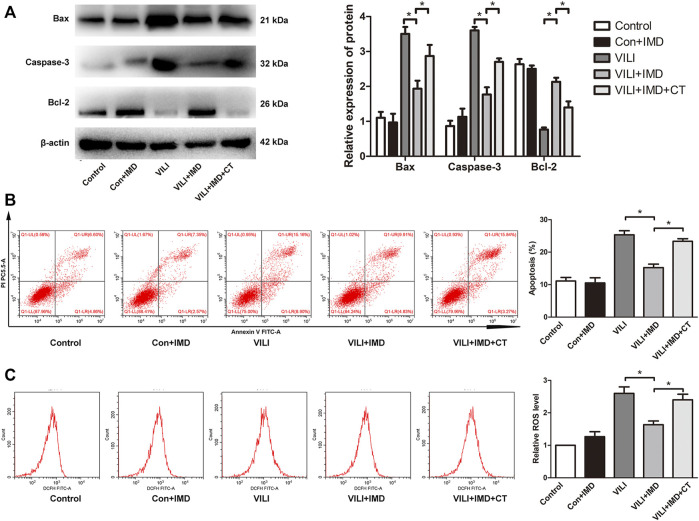
JAK2/STAT3 pathway was involved in IMD-mediated protection. **(A)** Western blot analysis of p-JAK2, JAK2, p-STAT3, STAT3 and β-actin in lung tissues. **(B)** Cell apoptosis rate was detected using flow cytometry. **(C)** Cell ROS was detected using flow cytometry. *p < 0.05.

To further clarify the participation of the JAK2/STAT3 signaling in VILI, the phosphorylation of JAK2 and STAT3 was measured by western blotting. In both mice and ECs, compared with the control group, the phosphorylation of JAK2 and STAT3 in the VILI group was significantly higher, and IMD reduced VILI-induced phosphorylation of JAK2 and STAT3 (Figures 4A,B). Future, we used Colivelin TFA (CT), an activator of the JAK2/STAT3 signaling pathway, to explore whether JAK2/STAT3 was involved in the IMD-mediated protective effects on VILI. Western blotting showed that in tissues, compared with the VILI + IMD group, the expression of Bax and caspase-3 increased significantly, and the expression of Bcl-2 declined significantly in the VILI + IMD + CT group (Figure 5A). In ECs, Flow cytometry results suggested that the activator of the JAK2/STAT3 significantly up-regulated the level of cell apoptosis (Figure 5B). We also found that CT partially reversed the antioxidant stress effects of IMD on VILI, manifesting in up-regulating EC ROS (Figure 5C). These findings suggested that IMD exerted anti-apoptotic and anti-oxidant stress effects via inhibiting JAK/STAT3 signaling.

## Discussion

**FIGURE 6:**
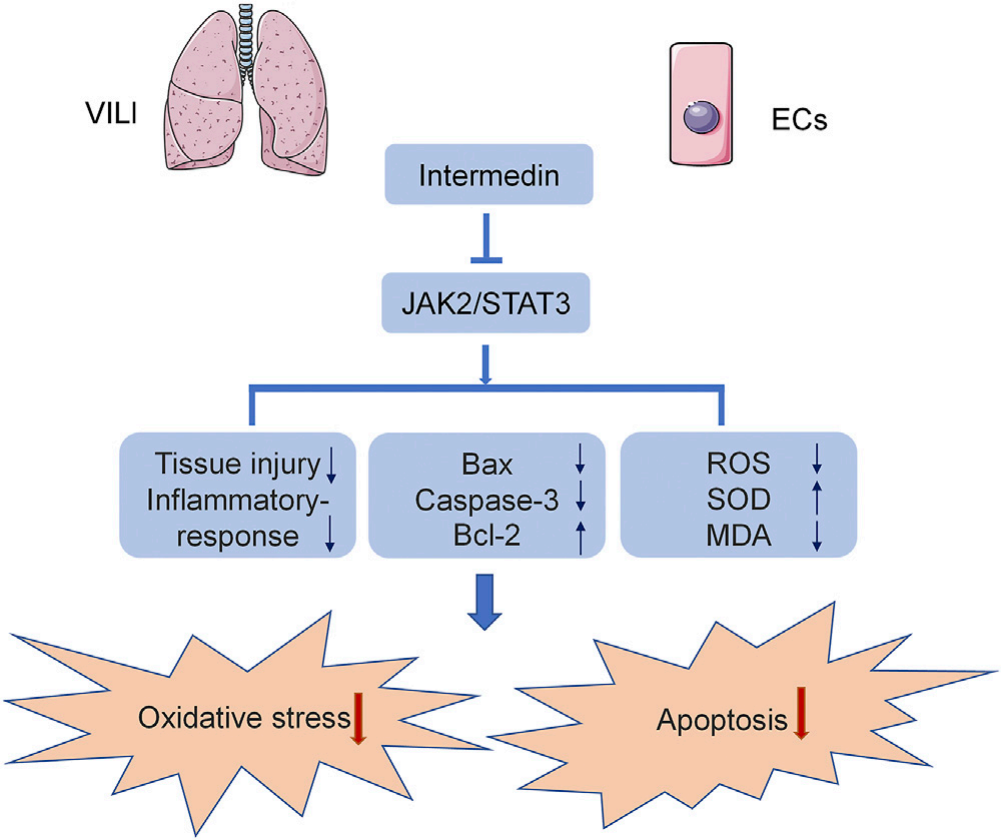
The diagram of mechanism we explored in the current study.

In the current study, we used C57B6/J mice and HPMECs to establish a VILI model to analyze the effect of IMD on VILI and its potential mechanism. We found, for the first time, that IMD improved lung injury and alleviated inflammatory factor expression in VILI. In addition, IMD also reduced oxidative stress by reducing ROS levels, upregulating SOD content, and downregulating MDA content. Western blot and immunohistochemical results showed that IMD also alleviated apoptosis, mainly by reducing the expression of Bax and caspase-3 and increasing the expression of Bcl-2. IMD exerted protective effects against VILI via JAK2/STAT3 signaling (Figure 6).

The IMD gene is located on human chromosome 22q13.33, and the encoded polypeptide chain is composed of 148 amino acids (Zhang and Xu, 2018). The N-terminal of IMD is an intramolecular ring composed of six amino acids and an α-helical structure (Takei et al., 2004; Holmes et al., 2020; Sohn et al., 2020). Human IMD sequences are 60% similar to fish and 87% similar to rodents (Roh et al., 2004). The high conservation of IMD also shows that it has important biological functions in the body. Through identification, it was found that IMD has three potential active cleavage fragments: IMD1-53 (Arg94-His95), IMD1-47 (Arg100-Thr101), and IMD1-40 (Arg107-Val108). The effects of different fragments may be different *in vivo*, and IMD1-53 has strong biological activity (Roh et al., 2004; Hirose et al., 2011; Zhu et al., 2016). IMD is widely expressed in the lung, heart, brain, kidney, and other tissues and organs, and produces various effects in the body through paracrine or autocrine processes. For example, IMD decreased orchitis in rats, alleviated macrophage aggregation and inflammatory cytokine levels in septic mice, and reduced acute organ injury (Li et al., 2013; Xiao et al., 2018). IMD also significantly eased chronic inflammation of adipose tissue by inhibiting AMPK phosphorylation and maintaining the balance of M1/M2 macrophages (Pang et al., 2016). In septic mice, IMD promoted the co-localization of Rab11 and VE-cadherin, repaired cell adhesion, and reduced vascular leakage (Xiao et al., 2018). Our previous studies have also shown that IMD reduced endothelial cell inflammation and improved the endothelial barrier integrity and functionality in LPS-induced ARDS (Fan et al., 2020). In the current study, we used IMD and IMD inhibitor to comprehensively confirm our hypothesis - IMD protected against VILI by alleviating oxidative stress and apoptosis. And we found that, on the one hand, compared with the VILI group, the VILI + IMD group had down-regulated inflammatory response, repaired lung injury, and reduced apoptosis and oxidative stress, suggesting that the administration of exogenous IMD had a protective effect on VILI. On the other hand, because IMD is widely expressed *in vivo*, we used IMD inhibitor to interfere the effect of endogenous IMD. Compared with the VILI group, the VILI + IMDinh group had more severe lung injury, more expression of inflammatory factors, and higher levels of apoptosis and oxidative stress. These results indicated that IMD inhibitor counteracted the protective effect of endogenous IMD. Overall, we confirmed the protective effect of IMD on VILI.

ECs are widely distributed throughout the body and have a large surface area. They mainly regulate the body’s homeostasis through six functions: regulating vasodilation-contraction, vascular permeability, cell growth, immunity and inflammation, coagulation function, cell growth, and lipid metabolism (Daiber et al., 2017; Fodor et al., 2021). Because the ability of endothelial cells to resist external stimulation is weak, endothelial cells are the firs to be affected and changed in acute lung injury (Wiener-Kronish et al., 1991; Matthay et al., 2019). EC activation may lead to mediator generation and leukocyte accumulation in the pulmonary capillary, further aggravating tissue injury. Studies have shown that EC dysfunction is mainly related to the decrease in the bioavailability of vasodilator substances (especially nitric oxide, NO) and the increase in vasoconstrictor substances (Lind et al., 2017; Cyr et al., 2020). However, excessive degradation or inactivation of NO caused by the increase in ROS is one of the main reasons for the decrease in NO bioavailability (Lind et al., 2017; Yepuri and Ramasamy, 2019). Upon injury, the production of ROS may be excessive, and the balance between oxidative stress and antioxidant stress is broken, EC function is destroyed, and apoptosis increases, which has a negative impact on cell and tissue function (Kuzkaya et al., 2003; Landmesser et al., 2003; Fodor et al., 2021). Therefore, it is essential to detect the oxidative and antioxidant stress functions of ECs in VILI. SOD is an antioxidant enzyme that scavenges superoxide anion free radicals and protects cells from damage caused by oxygen free radicals. MDA is a product formed by the reaction between lipids and oxygen free radicals, and its content represents the degree of lipid peroxidation (Abdelzaher et al., 2021). In the current study, we used frozen sections to detect the level of ROS in mouse lung tissue, flow cytometry to detect ROS content, and ELISA to detect the levels of SOD and MDA; we found that IMD could inhibit the oxidative stress in VILI. Consistent with our study, IMD alleviated oxidative stress and apoptosis in a NO-dependent manner in brain ECs (Chen et al., 2006). In cardiomyocytes, IMD inhibited oxidative stress and reduced ischemia-reperfusion injury through PI3K/Akt pathway (Song et al., 2009; Zhao et al., 2012).

The JAK2/STAT3 pathway is an intracellular signal transduction pathway that is involved in various biological processes and provides a direct mechanism for the regulation of gene expression by extracellular factors. Cytokines bind to receptors, resulting in receptor-coupled JAK aggregation and the activation of adjacent JAKs through mutual phosphorylation. The activated JAKs phosphorylate the tyrosine site on the receptor, causing the receptor to generate a STAT binding region and then phosphorylate STATs (O'Shea et al., 2015; Johnson et al., 2018; Xiong et al., 2021). In a septic mouse model, blocking the JAK2/STAT3 signaling pathway significantly reduced the levels of HMGB1 and key cytokines and alleviated multi-organ injury (Hui et al., 2009; Xin et al., 2020). In addition, the JAK2/STAT3 pathway affects cell apoptosis by altering the levels of Bcl-2 and Bax and influencing oxidative stress (Li et al., 2017; Raut et al., 2021). Therefore, we hypothesized that IMD could reduce oxidative stress and apoptosis through the JAK2/STAT3 pathway and protected against VILI. Consistent with previous studies, in the current study, we found that IMD inhibited the phosphorylation of JAK2 and STAT3 in VILI. The agonist of JAK2/STAT3 significantly reversed the anti-apoptotic and antioxidant stress effects mediated by IMD. However, the effects of STAT3 on lung tissue were not dogmatic. Previous study found that vagus nerve stimulation played a protective role in acute respiratory distress syndrome by increasing STAT3 and regulating macrophage transformation (Li et al., 2021). The diverse functions of STAT3 may be related to the different mechanisms of STAT3 in different cells types.

However, this study had some limitations. We found a protective effect of IMD on VILI, which indicated that IMD may be a potential target for the prevention and treatment of VILI. However, its mechanism remains unclear. In addition, although EC injury is vital in VILI, the pathogenesis of VILI is complex and diverse, and other injury mechanisms should not be ignored. Basic and clinical follow-up studies are essential to further clarify the potential role of IMD in VILI.

In conclusion, we found for the first time that IMD reduced VILI through anti-apoptotic and antioxidant stress in mice and HPMECs. In addition, we found that the anti-apoptotic and antioxidant stress effects of IMD were mediated by the inhibition of the JAK2/STAT3 signaling pathway.

## Data Availability

The original contributions presented in the study are included in the article/Supplementary Material, further inquiries can be directed to the corresponding authors.
